# Annual Report of the Necrologist of the Alumni Association of Chicago Medical College

**Published:** 1871-05

**Authors:** 


### ANNUAL REPORT OF THE NECROLOGIST OF THE ALUMNI ASSOCIATION OF CHICAGO MEDICAL COLLEGE.

*To the President of the Alumni Association:*

The Necrologist regrets that he has been able to make but little progress in compiling memoirs of deceased Alumni.

So far as can be learned, eleven deaths have occurred among the graduates of our Alma Mater.

The names of the deceased are, J. C. Pratt, Lucien Ashley, J. M. Kendall, D. J. Allaban, E. B. Rockwell, W. H. Pulver, J. J. Samuels, George W. Wilson, J. F. Flemming, and M. N. Rust.

Of four of these no information has been obtained, as to where they settled, or who can give information about them, notwithstanding careful inquiry has been made.

Letters of inquiry have been written to acquaintances or friends of all the rest, but answers, in the shape of any substantial statement of facts from which a memoir could be collated, have come back regarding only two of them, namely, of Dr. John Culbert Pratt and Dr. Lucien Ashley.

Such facts as have been gained have been written out and are here presented as a beginning of what it is hoped, with the co-operation of the members, may eventually be a complete, proper, and decent necrology of our Alumni.

If any graduate or friend of the school can give information or furnish any clue by which sketches of the remaining nine of the deceased Alumni can be written, if they will communicate it to the Necrologist, they will aid him materially in his labors, and help advance a work to all alike desirable.

It is quite esssential, however, that to get a biography of any man, in every particular correct, his business and professional associates must be consulted, and not merely his personal friends and family relatives, who, while giving dates and incidents rightly, are not always the most unbiased critics of a man's character.

What we want is unprejudiced histories, and they will confer a favor who can direct us to professional associates, as well as to relatives of these men.

Respectfully submitted,

N. BRIDGE, *Necrologist.*

CHICAGO, *March 14, 1871.*

---

DR. LUCIEN ASHLEY was born in East Barnard, Windsor County, Vermont, November 27th, 1833. His parents were descended from the early settlers of Massachusetts. He early evinced a love of books, and his childhood was spent in school; his youth in assisting in farm labors, alternating with attending school and teaching.

His health becoming impaired, in the Fall of 1854 he came to Illinois; the following Summer he spent in Minnesota. He returned in the Fall of '55, and commenced the study of medicine with his uncle, Dr. J. B. Ashley, of Magnolia, Putnam County, Illinois. In the Fall of '58 his name was entered as a student in the Chicago Medical College, and he graduated in the class of '60. He returned to Magnolia and commenced the practice of medicine in partnership with his uncle.

In the Fall of '61 he removed to Neponset, and three months later to Buda Illinois, where he soon acquired an extensive practice, and made many warm friends.

In the Spring of '63, during the seige of Vicksburg, Governor Yates issued a call for surgeons for the army. From the commencement of the war he had felt a strong desire to offer his services to his country, and he promptly responded to the Governor's call, contrary to the advice of friends, who much feared the effects of the change to that warm and unhealty climate, at that season of the year, on one in his feeble health. His reply to those who tried to dissuade him from going was, "If I live to return it will always be a satisfaction to me to think that I did what I could for my country in her hour of need; if I do not, I shall meet no worse fate than thousands of others."

He was assigned to the 3d Battallion, 10th Regiment, Illinois Cavalry. He joined the regiment on the 1st of June, and found them encamped six miles above Vicksburg, just outside the levee, on land that had been overflowed all the first part of the season. Half the men were on the sick list.

He remained on duty until the last latter of July, when he was attacked with dysentery, and died at Helena, Arkansas, on the 29th of that month.

Maj. Shaw, commanding the battalion, with the other officers and men, unanimously testified that "he served them faithfully and well as a medical officer, and profoundly regretted his early separation from them."

All his life he was remarkably conscientious and truthful, deeming no sacrifice too great if in the line of duty or right.

Of a modest and retiring disposition, "he bore acquaintance well," and those who knew him best loved him most.

He was ever a cherished associate, both at school and in later years, seeming to win all hearts by his kind and affectionate disposition; and in his professional career, rich and poor alike found him a kind and attentive physician and sympathizing friend.

He never made a profession of religion, but his private testimony, added to that of his life, warrants the statement that he was a sincere and consistent Christian.

John Culbert Pratt—Class of 1865.—Dr. Pratt was the second child of Mr. C. S. Pratt, a native of the Eastern States. His mother, whose maiden name was Rose Ann Culbert, although born in this country, was of Irish parentage.

The subject of this sketch was born June 5, 1835, in Nunda, then Alleghany County, New York.

When he was five years old the whole family removed to Vernon, Shiawassee County, Michigan, then a new and very thinly-settled place. Here he remained until he attained his majority.

Although naturally very studious, the common schools were, up to the twentieth year of his life, his only means of systematic instruction. Yet, at the age of eighteen, he had so far advanced in a knowledge of the common branches that he was able to teach such of the district schools as he had studied in. After two winters spent in this employment, he entered a school at Birmingham, Mich., under the tutorship of Prof. Samuel Hill; here he remained a full year, and was a diligent and an attentive student.

In the Autumn of 1856, a few months after his twenty-first birthday, he went to Illinois, and finding employment in a small school in the County of Williamson, he made there his temporary home. He continued in his vocation as teacher, in various schools, until the winter of 1859–60, when he was engaged by Dr. J. S. Jewell, since a professor in our Alma Mater, who was practising medicine in Williamson County, and who was a director in the school district in which he resided.

This acquaintance seems to have been the determining event in his career as a medical man, for while he was laboring daytimes in the school, he began studying medicine, spending his evenings with the library of Drs. Jewell and Mitchell. He studied early and late, and labored very hard during this early part of his term of medical study, and there can be little doubt that here he permanently injured his health. He pursued his medical studies diligently until the breaking out of the war.

Not long after the battle of Pittsburg Landing (Shiloh), when the government was in great need of medical and surgical attendants, for the wounded of that and subsequent engagements, Pratt entered the army as a contract surgeon.

It was during his service in the field that he contracted that disease which so decimated the ranks of the army, and from which he never fully recovered, chronic diarrhœa. Being unable to attend to duty, he was allowed to return home sometime during the early part of 1863.

With a change of climate and the kind offices of his friends, he recoverd in so far that he was anxious to return to the army. He was about to raise a company of volunteers, and go with them as captain, when Dr. Jewell persuaded him to go as assistant-surgeon instead. In this capacity he served eleven months, but his health again failed, and he was obliged to return to the North.

He went to his home in Michigan to recruit and recover, but so anxious was he to resume his medical studies, that in three weeks after his arrival he set out for Chicago. Here he became again the hard-working, attentive student. He attended lectures, and graduated with the class of 1865. In spite of ill-health he succeeded in gaining the first prize for the best thesis.

After his graduation he went back to Michigan, but his health did not improve, and his condition would not admit of his practicing, save to a very limited extent. His health gradually failed, and on the 19th of December, 1866, he died.

His disease—chronic diarrhœa—was of the most obstinate character. Dr. Jewell says his liver, and perhaps mesenteric glands, were seriously involved.

Dr. Pratt was never married.

Of his character and disposition I find no testimony but that in the highest degree commendatory. He was always thoughtful, considerate, and perhaps too generous and unselfish. Traits of this sort he manifested when very young; he was always ready to forego and forget his own pleasure and himself for his playmates; moreover, he was known to have made a favorite of a poor and ragged playmate, simply because he was poor and ragged.

. Of his character after attaining manhood, I must let one speak who knew him intimately, and whose words we regard. From a letter addressed me by Dr. J. S. Jewell, under date of October 6th, 1870, I read:

"I have known but few, if any, more estimable men than John C. Pratt; I knew him well; he was my favorite student and near friend. Handsome, frank, and manly in person and presence; cheerful and entertaining in manner; courteous, hopeful, and generous in disposition; decisive and upright in character; pure in intentions; rich in a warm, wise, and healthful sympathy; gifted with an excellent, well-balanced mind; independent in thought, but respectful; apt to teach, and ready to be taught; enterprising and inquiring; quick in perception; industrious; an excellent student, and giving unusual promise as a physician; a firm friend, true as steel, with warm emotions under excellent control; he was the one of a thousand.

"I have seldom felt more regret at the loss of one who was not a relative, and feel that the college roll has lost, in him, one of its best and brightest names, whose memory I trust will be sacredly cherished."

